# Boswellia serrata Enhances Passive Range-of-Motion Exercises in Radiation-Induced Trismus: A Case Report

**DOI:** 10.7759/cureus.58234

**Published:** 2024-04-14

**Authors:** Derek A Mumaw, Tracy M Nassif, Melissa A Witsil, Rohan L Deraniyagala

**Affiliations:** 1 Radiation Oncology, Beaumont Hospital, Royal Oak, USA; 2 Speech Pathology, Beaumont Hospital, Royal Oak, USA

**Keywords:** radiation therapy, supplement, therabite, boswellia serrata, trismus

## Abstract

Trismus is a common, extremely detrimental side effect following definitive radiotherapy for head and neck malignancies. Existing therapeutic modalities (active and passive range-of-motion exercises and systemic therapies) offer only modest, slow improvements in jaw opening; thus, there is a need for additional treatment options. *Boswellia serrata *(*BS*) ("Indian frankincense") is a tree native to West Asia and North Africa that produces resin-containing “boswellic” acids. These have been shown to have in vitro and in vivo anti-inflammatory effects and have previously been found to be an effective treatment for asthma, colitis, arthritis, and post-radiation edema. Herein we report the case of a 54-year-old male with severe post-radiation trismus who experienced a dramatic resolution with *BS*/Therabite® combination therapy. His trismus improved from 6 mm to 45 mm over 10 weeks (0.46 mm/day), far exceeding previous rates of improvement documented in the literature. There were no ill effects. Given the dearth of effective treatments for post-radiation trismus, *BS *is a promising agent deserving of further study.

## Introduction

Trismus's definition and impact

Trismus is a pathologic restriction in jaw opening, defined as an opening of ≤35 mm, typically caused by fibrosis following surgery and/or radiation or more rarely as a result of direct tumor involvement of the masticatory muscles [1]. It is a common problem in head and neck cancer treatment: approximately 50% of patients who undergo definitive treatment involving radiation therapy will meet the Djikstra definition for trismus; oropharyngeal disease is particularly prone with nearly two-thirds meeting the definition [2,3]. A meta-analysis noted a 44% prevalence at six months falling to 32% at 12 months and remaining stable thereafter [4]. Not only is trismus common but it also substantially degrades the quality of life with significant detriments to mouth opening, chewing, food intake, and overall quality of life [3,5].

Existing therapies and effectiveness

There are a number of management options for patients experiencing trismus. Perhaps most important is physical therapy, which plays an integral role in the management of trismus with interventions including both passive and active range-of-motion exercises as well as electrotherapy. Passive range-of-motion exercises are often performed using devices that provide a controlled opening force to the jaw. These can be ad-hoc as in stacked tongue depressors or wooden spatulas or commercially produced as the Therabite® and Dynasplint® systems [6,7].

The effectiveness of these commercially produced devices in reducing trismus has been assessed in a number of studies. The literature regarding Dynasplint® has shown overall effect sizes ranging from 7 mm to 13.6 mm of increased jaw opening with a rate of change estimate of 0.36 mm per day [6,8,9]. Regarding Therabite®, Sherpenhuizen et al. performed a systematic review that identified four studies assessing the impact of this device on radiotherapy-induced trismus [10]. Kamstra et al. identified a 5.4 mm mean increase and Tang et al. showed a net mean benefit of 5 mm [11,12]. Of particular interest are Buchbinder et al. and Pauli et al., which also report the duration of Therabite® treatment allowing for a calculation of the rate of improvement: 0.19 mm/day and 0.09 mm/day, respectively [13,14].

Some systemic therapies have shown promise in augmenting physical therapy and device-based interventions, notably pentoxifylline, which has been shown to exert only a modest benefit with a mean improvement of 4 mm [15]. Botulinum toxin has also been tested. Though it was found to improve pain and masticator spasms, it provides no direct benefit to jaw opening [16]. Despite all of these therapeutic modalities, trismus treatment remains characterized by slow, modest gains.

Boswellia serrata

*Boswellia serrata* (*BS*) is a tree native to North Africa, the Middle East, and India [17]. It produces a natural resin known colloquially as "Indian frankincense" and "salai," which has long been used as an incense and aromatic. It also has found use in traditional Ayurvedic medicine where it is considered to be an effective antirheumatic agent. The resin is a complex amalgam of molecules but uniquely contains pentacyclic triterpenic acids known as "boswellic acids" [18]. These molecules have been found to have anti-inflammatory properties through their inhibition of 5-lipoxygenase, human leukocyte elastase, TNF-α, interleukin-1β, NF-κB, VEGF, and TGF-β [19-24].

These in vitro anti-inflammatory effects have translated to in vivo benefits. One of the first studies was in asthma: Gupta et al. performed a double-blind, randomized controlled trial where *BS* was shown to result in 70% symptomatic improvement compared to 27% in the control arm [25]. Gerhardt et al. showed *BS *to be non-inferior to mesalazine in Crohn's disease and Madisch et al. showed an absolute 37% increase in the rate of clinical remission of collagenous colitis [26,27]. Kimmatkar et al. and Thawani et al. both performed randomized controlled trials of *BS *in osteoarthritis and found improvements in pain, movement, swelling, and ability to accomplish activities of daily living [28,29]. Most recently, there has been a surge of interest in *BS *in the setting of post-radiation edema following partial-brain and whole-brain radiation therapy. Retrospective reviews, case reports, and case series have documented a substantial reduction in cerebral edema with *BS *[30-32]. Kirste et al. performed a randomized controlled trial that demonstrated a >75% reduction in cerebral edema in 60% of patients receiving *BS *(compared to only 26% of those receiving a placebo) [33].

In these studies, the toxicity of *BS *was minimal and mild. None of the studies discussed here report any serious adverse events. Most reported mild non-specific gastrointestinal symptoms: Madisch et al. reported one patient (6.25%) with mild dizziness, hypoglycemia, and lack of appetite and another who developed persistent diarrhea and bacterial enteritis; Kimmatkar et al. reported one patient (3%) with loose bowel movements and another developed epigastric pain and nausea; Thawani et al. reported one patient (3%) with diarrhea and abdominal cramps and three patients with reflux; finally, Kirste et al. reported six patients (27%) with grade 1-2 diarrhea [27-29,33].

To our knowledge, there have been no reports documenting the use of *BS *in treating trismus. Herein, we provide a case report of *BS *augmenting passive range-of-motion interventions leading to the rapid resolution of the patient's trismus.

## Case presentation

**Figure 1:**
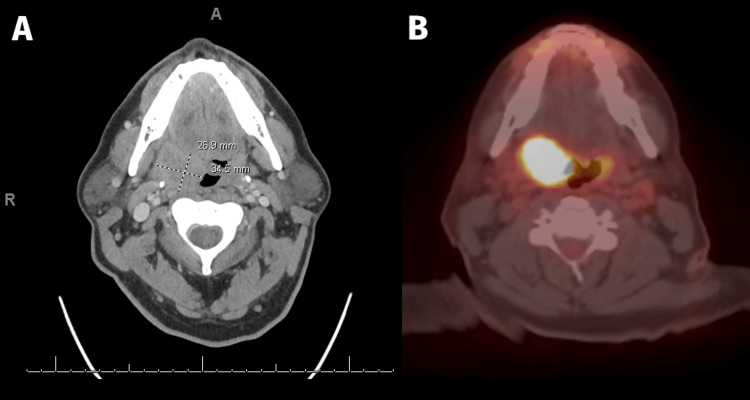
Diagnostic imaging of the primary site. Diagnostic CT (A) and PET/CT (B) imaging showing an FDG-avid 27x35x42 mm mass in the right palatine tonsil.

Our patient is a 54-year-old previously healthy male with no relevant past medical history. He had a two-pack per year smoking history from 1990 to 2013 (~3-4 cigarettes per day) followed by weekly cigar use until 2020 at which point it became daily. He initially presented with discomfort and a sensation of fullness in the right posterolateral oropharynx with mild right-sided trismus. This prompted a referral to an oral surgeon who ordered a contrast-enhanced CT of the neck showing a 4.2 cm homogeneously enhancing mass of the right palatine tonsil abutting the pterygoid musculature; there was no evidence of any regional nodal disease. He was referred to an otolaryngologist at an outside institution who performed a biopsy revealing p16-positive squamous cell carcinoma. A staging PET/CT was then performed, demonstrating hypermetabolism of the biopsy-proven right tonsillar mass that extended inferiorly, effacing the right vallecula. There was no evidence of hypermetabolic regional or distant disease. His imaging is shown in Figure 1.

**Figure 2:**
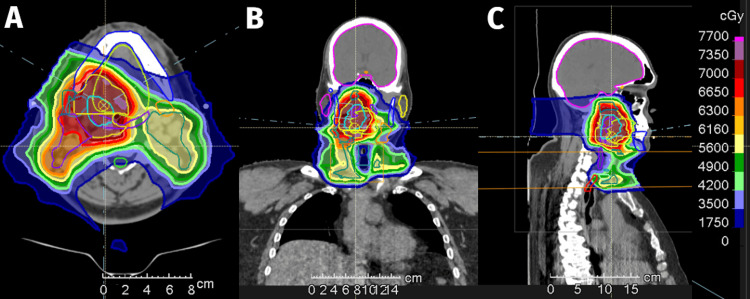
Treatment plan delivered. Representative axial (A), coronal (B), and sagittal (C) sections of the proton dose distribution delivered to the patient.

**Figure 3:**
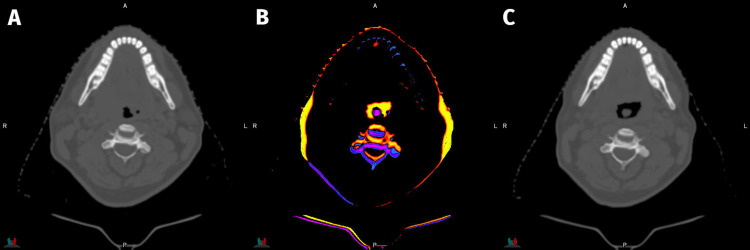
Weight loss during treatment prompting replanning. During the treatment course, the patient was found to have substantial weight loss compared to his initial simulation CT (A). This is reflected in the repeat CT simulation (C). The middle pane (B) shows a difference plot with the yellow-green showing substantial loss of fat tissue laterally.

For this, the patient underwent definitive chemoradiation therapy with 70 Gy relative biologic equivalent (RBE) delivered to gross disease, 63 Gy (RBE) to high-risk surrounding tissue, and 56 Gy (RBE) of elective bilateral nodal irradiation (levels II-IV) all in 35 fractions via a simultaneous integrated boost as depicted in Figure 2. Protons were used to minimize toxicity in the setting of bilateral elective neck treatment. Weekly cisplatin was administered concurrently. Treatment was completed in 50 days without any unanticipated complications or significant delays. A single replan was required due to tumor response and weight loss noted on cone beam CT (Figure 3). The patient experienced expected mild-to-moderate fatigue, mucositis, odynophagia, and dermatitis during his course of treatment.

At the time of the patient's three-month post-treatment follow-up, he noted the development of mild bilateral cervical and submental lymphedema; however, much of the acute, treatment-related side effects had resolved with only mild dysgeusia and mild trismus (22 mm) remaining. Over the course of the following three weeks, the patient's trismus dramatically worsened, nadiring at 4-6 mm. Passive range-of-motion exercises using a custom-made tongue depressor fulcrum device were started at this point with minimal improvement. The patient met with his otolaryngologist who recommended referral for coronoidectomy. Motivated by a desire to avoid surgery, the patient began treatment with the Therabite® system. Two days later, the patient began treatment with *BS* (4.5 g/day, split between three doses). This dose was chosen as it was the most common dosing regimen used in recent studies treating radiation-induced adverse effects [30,32,33].

**Figure 4:**
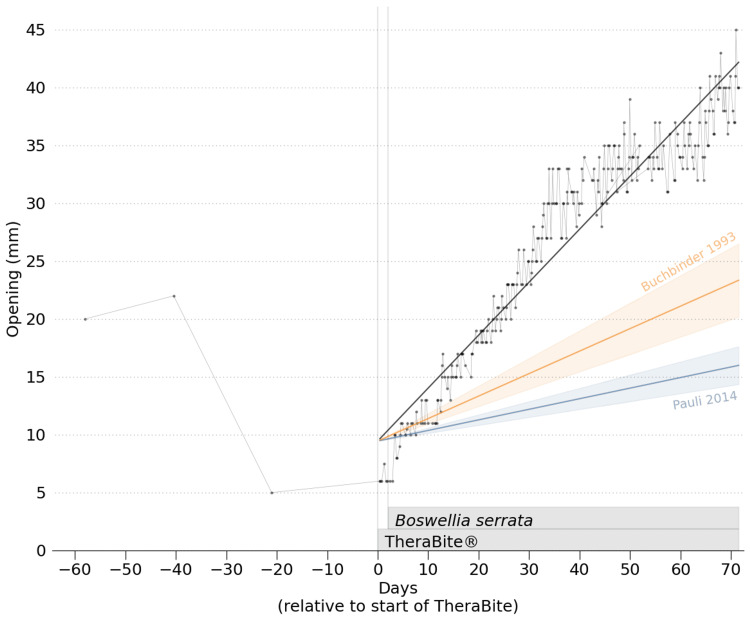
Trismus development and resolution timeline. Timeline of trismus development and resolution with *BS* and Therabite® start dates. Trismus resolution rates (with 95% confidence intervals) from Buchbinder et al. (orange) and Pauli et al. (blue) are superimposed. Dates are relative to the start of Therabite® [13,14].

The treatment response is documented in Figure 4 with day 21 representing his jaw-opening nadir, day 0 representing his initiation of Therabite®, day 2 representing his initiation of *BS*, and the subsequent improvement thereafter with records ending at day 72. At that point, he had achieved a net improvement in jaw opening of 39 mm, consistent with a 0.46 mm/day rate of improvement. Within 1 week of starting BS, the patient also noted resolution of his cervical/submental lymphedema, gingival edema, and improvement in his chronic bilateral knee osteoarthritis, which was maintained until the time of his five-month post-treatment follow-up visit (the most recent encounter with the patient). The patient experienced no side effects, which he could attribute to *BS*.

Patient experience

“My trismus started to develop two months after my radiation treatment ended. I started taking muscle relaxers and stretching with stacked tongue depressors and a homemade tongue depressor fulcrum device, but my condition progressively got worse. I was seeing a physical therapist for lymphedema but for the trismus, all they could do was a light massage. After a month of this, I decided to purchase the Therabite® but I had difficulty with it because I couldn't open my mouth wide enough to use it. I was having to stretch with the tongue depressors first before I could even begin with the Therabite® and by then I was in extreme pain and couldn't go on. I became really discouraged and just went back to stretching for another few weeks with the tongue depressors hoping things would get better enough to start using the Therabite® again. All the while I continued to lose more weight on top of the weight I lost during treatment. In the meantime, I had an appointment with my ENT who explained that the longer this condition lasts the worse it will get and suggested a consult with an oral surgeon for the possibility of surgery. After talking to my radiation oncologist and telling him about the consult, he suggested I give *Boswellia *a try before the surgery option.

I was willing to try anything after two months of stretching and taking muscle relaxers with no real progress. I stopped taking the muscle relaxers and purchased the *Boswellia, *which was easy to find [an online retailer]. I also started using the Therabite® again along with pre-stretching with stacked tongue depressors. Two days later the *Boswellia *arrived, and I started taking it. Within a week I went from 6 mm before stretching to 10 mm and no longer needed to pre-stretch with the tongue depressors. My condition continued to improve to where I was able to chew small pieces of food again within two weeks. Within two months, I reached 35 mm before stretching.

My experience with *Boswellia *has been amazing. I believe being able to take something that can attack the problem from the inside in addition to Therabite® stretching was the difference. There was no downside to it. I experienced no bad side effects and in fact the knee pain I was experiencing disappeared. Also, the swelling and inflammation in my tonsil and gums behind my back molar had not changed in the three months since my treatment ended. After taking *Boswellia*, all the swelling and inflammation were gone in a matter of a couple of weeks. I was also able to stabilize my weight loss, which was becoming a problem.” 

Quoted directly from the patient, modified only to ensure proper rendering of "Therabite®" and "Boswellia" and to remove other branding.

## Discussion

As described above, here we have a patient with severe trismus who, after initially struggling with passive range-of-motion exercises (achieving only 1-2 mm of opening), had a dramatic response to combination therapy with Therabite® and *BS*. Over the course of 72 days, the patient improved his jaw opening from 6 mm to 45 mm: an impressive gain of 39 mm or 0.46 mm/day.

This is far faster than that previously documented in the Therabite® literature: 5.0 times faster than the rate determined by Pauli et al. and 2.4 times faster than Buchbinder et al. [13,14]. Even the best-case scenario, the upper limit of Buchbinder's 95% confidence interval, is 1.9x lower than our patient. Our patient's rate of improvement lies an impressive 2.5 standard deviations above Buchbinder's mean.

It seems difficult, then, to attribute the patient's rapid improvement solely to his use of the Therabite® system. Within two days of initiating Therabite®, the patient also began taking *BS*. As mentioned, *BS *has a well-documented anti-inflammatory effect, with in vitro and in vivo studies both identifying several targets of activity including 5-lipoxygenase, human leukocyte elastase, TNF-α, interleukin-1β, NF-κB, VEGF, and TGF-β [19-24]. These molecular targets are of particular interest in the post-radiation setting in that they are known to mediate the continuous inflammatory state, caused by persistent oxidative stress and hypoxia, ultimately leading to fibrosis and necrosis [34-39]. TGF-β has been specifically implicated in the etiopathogenesis of radiation-induced trismus by inducing myofibroblast differentiation and subsequently stimulating their secretion of collagen, fibronectin, and proteoglycans as well as by downregulating matrix metalloproteinase activity. Thus, the rate of extracellular matrix deposition is increased while its degradation is inhibited, leading to the stiffening of tissue [40]. Inhibition of these pro-inflammatory, pro-fibrotic pathways may be the mechanism through which *BS* is augmenting the speed of the patient's recovery.

We also see several other systemic effects that support this anti-inflammatory hypothesis. Within a week of initiating treatment with *BS*, the patient noted a substantial improvement in his gingival edema, cervical lymphedema, and arthritic pain. These, of course, cannot be explained by the Therabite®, and the abruptness and concurrence of the recovery for each issue is inconsistent with a spontaneous resolution. This is, again, suggestive of a strong anti-inflammatory effect with *BS*.

When considering the added value of any new therapy, it is also important to consider its side effects. In the case of *BS*, there are no documented serious adverse effects in the literature, only mild gastrointestinal symptoms like nausea and diarrhea. This stands in contrast to the other available systemic agents for trismus: pentoxifylline is associated with gastrointestinal (discomfort, bloating, and diarrhea), neurologic (dizziness and headache), and cardiovascular (flushing, arrhythmias, etc.) side effects while botulinum toxin may cause injection-site pain, edema, erythema, ecchymosis, and hypo/hypesthesia as well as more serious side effects such as muscle spasms, headaches, denervation, and dysphagia [41]. Our case report suggests that *BS *is both more effective and less side effect prone than these.

## Conclusions

Given the extremely detrimental effects of trismus on quality of life and the slow, modest gains effected by the current suite of treatments, there is an unmet need for additional therapies. We believe that BS may meet this need, at least in part, as evidenced by our patient's dramatic recovery of jaw mobility. Though this is the first documented use in the setting of trismus, it is consistent with the literature, which has reported promising results in several disease processes while carrying a minimal risk of mild side effects. Further studies are needed to explore the role of BS in the treatment of trismus, and prospective studies may be justified to quantify its benefit.
